# Supplementary material for: Unveiling the Bioactive Potential of the Invasive Jellyfish Phyllorhiza punctata Through Integrative Transcriptomic and Proteomic Analyses
Source: Biomolecules. 2025 Aug 4;15(8):1121. doi: 10.3390/biom15081121 (PMC12383608; doi:10.3390/biom15081121)
Supplement: Supplementary file 1 [file biomolecules-15-01121-s001.zip › Suplementary Material/FileS3/PEPFOLD4/XP_065059349_5567/clusters.html]

 PEP-FOLD clusters


Download archive of all models  here   

|  |  |  |
| --- | --- | --- |
| # | file | sOPEP |
| # cluster 1 - Size: 6 |  |  |
|  | XP\_065059349\_5567-model1 | -14.6734 |
|  | XP\_065059349\_5567-model1.1 | -14.5128 |
|  | XP\_065059349\_5567-model1.2 | -14.5128 |
|  | XP\_065059349\_5567-model1.3 | -14.5128 |
|  | XP\_065059349\_5567-model1.4 | -14.5128 |
|  | XP\_065059349\_5567-model1.5 | -14.4805 |
| # cluster 2 - Size: 36 |  |  |
|  | XP\_065059349\_5567-model2 | -14.6567 |
|  | XP\_065059349\_5567-model2.1 | -14.6567 |
|  | XP\_065059349\_5567-model2.2 | -14.6567 |
|  | XP\_065059349\_5567-model2.3 | -14.6567 |
|  | XP\_065059349\_5567-model2.4 | -14.6567 |
|  | XP\_065059349\_5567-model2.5 | -14.6567 |
|  | XP\_065059349\_5567-model2.6 | -14.6567 |
|  | XP\_065059349\_5567-model2.7 | -14.6567 |
|  | XP\_065059349\_5567-model2.8 | -14.6567 |
|  | XP\_065059349\_5567-model2.9 | -14.6567 |
|  | XP\_065059349\_5567-model2.10 | -14.5174 |
|  | XP\_065059349\_5567-model2.11 | -14.5174 |
|  | XP\_065059349\_5567-model2.12 | -14.5174 |
|  | XP\_065059349\_5567-model2.13 | -14.5072 |
|  | XP\_065059349\_5567-model2.14 | -14.5072 |
|  | XP\_065059349\_5567-model2.15 | -14.5072 |
|  | XP\_065059349\_5567-model2.16 | -14.5072 |
|  | XP\_065059349\_5567-model2.17 | -14.5072 |
|  | XP\_065059349\_5567-model2.18 | -14.5072 |
|  | XP\_065059349\_5567-model2.19 | -14.5072 |
|  | XP\_065059349\_5567-model2.20 | -14.5072 |
|  | XP\_065059349\_5567-model2.21 | -14.5072 |
|  | XP\_065059349\_5567-model2.22 | -14.5072 |
|  | XP\_065059349\_5567-model2.23 | -14.5072 |
|  | XP\_065059349\_5567-model2.24 | -14.5072 |
|  | XP\_065059349\_5567-model2.25 | -14.5072 |
|  | XP\_065059349\_5567-model2.26 | -14.5072 |
|  | XP\_065059349\_5567-model2.27 | -14.5072 |
|  | XP\_065059349\_5567-model2.28 | -14.5072 |
|  | XP\_065059349\_5567-model2.29 | -14.5072 |
|  | XP\_065059349\_5567-model2.30 | -14.5072 |
|  | XP\_065059349\_5567-model2.31 | -14.5072 |
|  | XP\_065059349\_5567-model2.32 | -14.5072 |
|  | XP\_065059349\_5567-model2.33 | -14.3803 |
|  | XP\_065059349\_5567-model2.34 | -14.3779 |
|  | XP\_065059349\_5567-model2.35 | -14.3779 |
| # cluster 3 - Size: 17 |  |  |
|  | XP\_065059349\_5567-model3 | -14.6123 |
|  | XP\_065059349\_5567-model3.1 | -14.5183 |
|  | XP\_065059349\_5567-model3.2 | -14.5183 |
|  | XP\_065059349\_5567-model3.3 | -14.5183 |
|  | XP\_065059349\_5567-model3.4 | -14.5183 |
|  | XP\_065059349\_5567-model3.5 | -14.5183 |
|  | XP\_065059349\_5567-model3.6 | -14.5183 |
|  | XP\_065059349\_5567-model3.7 | -14.5183 |
|  | XP\_065059349\_5567-model3.8 | -14.5183 |
|  | XP\_065059349\_5567-model3.9 | -14.4714 |
|  | XP\_065059349\_5567-model3.10 | -14.4714 |
|  | XP\_065059349\_5567-model3.11 | -14.4714 |
|  | XP\_065059349\_5567-model3.12 | -14.4714 |
|  | XP\_065059349\_5567-model3.13 | -14.4714 |
|  | XP\_065059349\_5567-model3.14 | -14.4714 |
|  | XP\_065059349\_5567-model3.15 | -14.4714 |
|  | XP\_065059349\_5567-model3.16 | -14.4714 |
| # cluster 4 - Size: 7 |  |  |
|  | XP\_065059349\_5567-model4 | -14.5723 |
|  | XP\_065059349\_5567-model4.1 | -14.5723 |
|  | XP\_065059349\_5567-model4.2 | -14.5723 |
|  | XP\_065059349\_5567-model4.3 | -14.5723 |
|  | XP\_065059349\_5567-model4.4 | -14.5723 |
|  | XP\_065059349\_5567-model4.5 | -14.4975 |
|  | XP\_065059349\_5567-model4.6 | -14.3705 |
| # cluster 5 - Size: 4 |  |  |
|  | XP\_065059349\_5567-model5 | -14.5511 |
|  | XP\_065059349\_5567-model5.1 | -14.5511 |
|  | XP\_065059349\_5567-model5.2 | -14.4799 |
|  | XP\_065059349\_5567-model5.3 | -14.4799 |
| # cluster 6 - Size: 8 |  |  |
|  | XP\_065059349\_5567-model6 | -14.5306 |
|  | XP\_065059349\_5567-model6.1 | -14.5192 |
|  | XP\_065059349\_5567-model6.2 | -14.5192 |
|  | XP\_065059349\_5567-model6.3 | -14.5192 |
|  | XP\_065059349\_5567-model6.4 | -14.4973 |
|  | XP\_065059349\_5567-model6.5 | -14.4973 |
|  | XP\_065059349\_5567-model6.6 | -14.4973 |
|  | XP\_065059349\_5567-model6.7 | -14.4048 |
| # cluster 7 - Size: 15 |  |  |
|  | XP\_065059349\_5567-model7 | -14.5218 |
|  | XP\_065059349\_5567-model7.1 | -14.5218 |
|  | XP\_065059349\_5567-model7.2 | -14.5 |
|  | XP\_065059349\_5567-model7.3 | -14.5 |
|  | XP\_065059349\_5567-model7.4 | -14.5 |
|  | XP\_065059349\_5567-model7.5 | -14.5 |
|  | XP\_065059349\_5567-model7.6 | -14.5 |
|  | XP\_065059349\_5567-model7.7 | -14.5 |
|  | XP\_065059349\_5567-model7.8 | -14.5 |
|  | XP\_065059349\_5567-model7.9 | -14.5 |
|  | XP\_065059349\_5567-model7.10 | -14.5 |
|  | XP\_065059349\_5567-model7.11 | -14.5 |
|  | XP\_065059349\_5567-model7.12 | -14.5 |
|  | XP\_065059349\_5567-model7.13 | -14.4843 |
|  | XP\_065059349\_5567-model7.14 | -14.4304 |
| # cluster 8 - Size: 5 |  |  |
|  | XP\_065059349\_5567-model8 | -14.5052 |
|  | XP\_065059349\_5567-model8.1 | -14.5052 |
|  | XP\_065059349\_5567-model8.2 | -14.5052 |
|  | XP\_065059349\_5567-model8.3 | -14.5052 |
|  | XP\_065059349\_5567-model8.4 | -14.4508 |
| # cluster 9 - Size: 1 |  |  |
|  | XP\_065059349\_5567-model9 | -14.3844 |
| # cluster 10 - Size: 1 |  |  |
|  | XP\_065059349\_5567-model10 | -14.34 |
|  |  |  |
